# Obicetrapib and lipoprotein(a) levels in patients at high cardiovascular risk: a pooled analysis of trials

**DOI:** 10.1093/eurheartj/ehag399

**Published:** 2026-05-25

**Authors:** Stephen J Nicholls, Adam J Nelson, Kausik K Ray, Marc Ditmarsch, Douglas Kling, Andrew Hsieh, Michael Szarek, John J Kastelein, Michael H Davidson

**Affiliations:** Victorian Heart Institute, Monash University, 631 Blackburn Road, Clayton VIC 3168, Australia; Victorian Heart Institute, Monash University, 631 Blackburn Road, Clayton VIC 3168, Australia; School of Public Health, Imperial College London, London, UK; NewAmsterdam Pharma, Amsterdam, The Netherlands; NewAmsterdam Pharma, Amsterdam, The Netherlands; NewAmsterdam Pharma, Amsterdam, The Netherlands; University of Colorado Anschutz Medical Campus, Aurora, CO, USA; State University of New York Downstate School of Public Health, Brooklyn, NY, USA; NewAmsterdam Pharma, Amsterdam, The Netherlands; Department of Vascular Medicine, University of Amsterdam, Amsterdam, The Netherlands; NewAmsterdam Pharma, Amsterdam, The Netherlands

**Keywords:** Cholesteryl ester transfer protein, Cardiovascular risk, Lipoprotein(a), Prevention

## Abstract

**Background and Aims:**

There is interest in developing lipoprotein(a) [Lp(a)] lowering therapies. Cholesteryl ester transfer protein inhibitors lower Lp(a), however the effects of the selective inhibitor obicetrapib on Lp(a) levels in high cardiovascular risk patients have not been fully elucidated. This analysis investigated the effect of obicetrapib on Lp(a).

**Methods:**

A pooled analysis of trials evaluating the 12-week lipid effects of daily obicetrapib 10 mg compared with placebo in patients with heterozygous familial hypercholesterolemia (HeFH) or atherosclerotic cardiovascular disease (ASCVD) investigated the effect of obicetrapib on Lp(a) levels.

**Results:**

In 2356 patients, the pooled cohort had a median age of 66 years, 36% were female with a history of ASCVD in 82%, HeFH in 27% and statin use in 91%. Median baseline lipid levels included low-density lipoprotein cholesterol (LDL-C) of 92 mg/dL, apolipoprotein B (apoB) of 87 mg/dL and Lp(a) of 42.9 nmol/L. Obicetrapib produced placebo-adjusted reductions in LDL-C of 37.0% and 35 mg/dL, in apoB of 21.3% and 20 mg/dL, and in Lp(a) of 37.3% and 14.9 nmol/L. In patients with baseline Lp(a) levels ≥50-<150 nmol/L, obicetrapib produced placebo-adjusted reductions in Lp(a) of 43.3% and 36.3 nmol/L. While patients with baseline Lp(a) ≥150 nmol/L demonstrated a lower percentage reduction in Lp(a) with obicetrapib than those with baseline levels ≥50-<150 nmol/L, the absolute reduction in Lp(a) was similar in both groups (−32.3 vs −36.3 nmol/L).

**Conclusions:**

Obicetrapib lowered LDL-C, apoB and Lp(a). The absolute reduction in Lp(a) with obicetrapib was similar in patients with mildly elevated Lp(a) levels, who are unlikely to qualify for administration of RNA-targeted Lp(a) lowering agents.

## Structured Graphical Abstract

**Structured graphical abstract:**
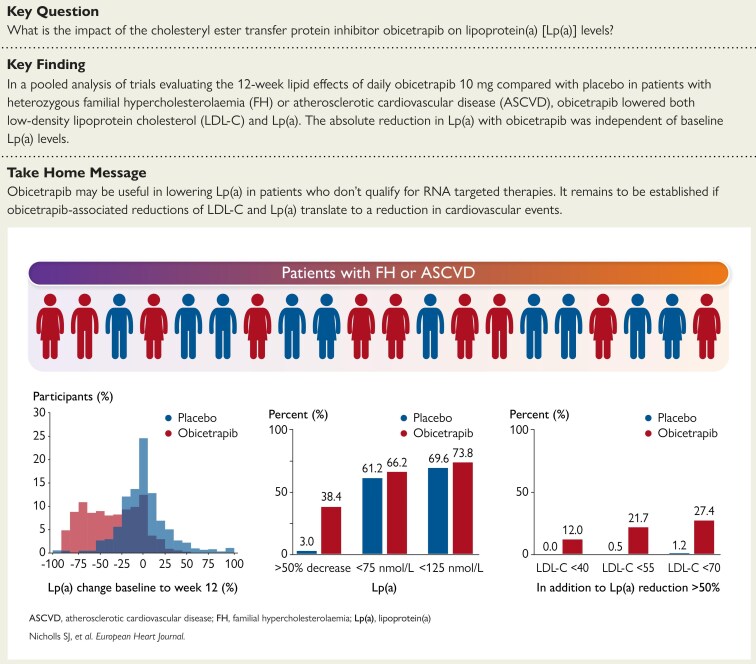

## Introduction

Increasing evidence implicates lipoprotein(a) [Lp(a)] in the causality of cardiovascular disease. Preclinical studies have demonstrated functional properties of Lp(a) that extend beyond its role as an apolipoprotein B (apoB) containing particle that promotes foam cell formation, including adverse effects on inflammatory, thrombotic and calcific pathways.^1^ Genomic studies have demonstrated that Lp(a) is more atherogenic than low-density lipoproteins (LDL)^2^ and that lowering Lp(a) has the potential to reduce cardiovascular risk.^3,4^ This is supported by observations that Lp(a) lowering with proprotein convertase subtilisin kexin type 9 (PCSK9) inhibitors independently associates with their ability to reduce cardiovascular event rates.^5,6^ Accordingly, there is considerable interest in the development of novel therapeutic approaches to lowering Lp(a) levels.

Cholesteryl ester transfer protein (CETP) plays an important role in lipid metabolism, facilitating transfer of esterified cholesterol from high-density lipoproteins (HDL) to LDL and very low-density lipoproteins in exchange for triglycerides.^7^ While early development of CETP inhibitors was stimulated by their ability to raise HDL cholesterol (HDL-C), more recent efforts have focussed on their role in LDL cholesterol (LDL-C) lowering. These agents have also been demonstrated to lower Lp(a) levels. Obicetrapib is a highly selective CETP inhibitor, which produces robust LDL-C lowering and HDL-C raising in patients treated with maximally tolerated statin therapy.^8–11^ Pooled analyses of early clinical trials of obicetrapib have reported a trend towards less cardiovascular events within the first 12 months of treatment.^12^ This analysis aimed to determine the effects of obicetrapib on Lp(a) levels in a pooled analysis of two clinical trials evaluating its lipid effects with 12 months of treatment.

## Methods

### Study design

This analysis investigated changes in Lp(a) concentration in two phase 3 clinical trials evaluating the lipid-lowering effects of obicetrapib in patients at high risk of cardiovascular events. The Evaluate the Effect of Obicetrapib in Patients with HeFH on Top of Maximum Tolerated Lipid-Modifying Therapies (BROOKLYN) study (NCT05425745) was conducted to determine the degree of LDL-C reduction with addition of obicetrapib in patients treated with heterozygous familial hypercholesterolaemia (HeFH). The study recruited 354 adults at least 18 years of age, with HeFH diagnosed by genetic confirmation, Dutch Clinical Network score or Simon Broome criteria, treated with maximally tolerated lipid-lowering therapy, who had evidence of suboptimal lipid control, defined by the presence of a LDL-C at least 70 mg/dL. Exclusion criteria included a cardiovascular event in the prior 3 months, homozygous familial hypercholesterolaemia, uncontrolled hypertension or diabetes, active liver disease or history of malignancy. Eligible patients were randomized (2:1) in permuted blocks to treatment with obicetrapib 10 mg or matching placebo, administered orally daily for 365 days. The primary endpoint was the per cent change in LDL-C from baseline to day 84. Secondary endpoints included the change in LDL-C from baseline to day 365, changes in other lipid and lipoprotein parameters and the per cent of patients achieving various LDL-C goals.

The Randomized Study to Evaluate the Effect of Obicetrapib on Top of Maximum Tolerated Lipid-Modifying Therapies (BROADWAY, NCT05142722) was conducted to determine the effect of obicetrapib on lipid levels and characterize its safety and tolerability in patients at high risk of cardiovascular events. The study recruited 2530 adults at least 18 years of age, with high cardiovascular risk determined by the presence of either HeFH or atherosclerotic cardiovascular disease (ASCVD), treated with maximally tolerated lipid-lowering therapy, who had evidence of suboptimal lipid control, defined by the presence of a LDL-C at least 100 mg/dL or non-HDL-C at least 130 mg/dL or in the presence of at least one enhancing risk factor a LDL-C between 55 and 100 mg/dL or non-HDL-C between 85 and 130 mg/dL. Exclusion criteria included a cardiovascular event in the prior 3 months, homozygous familial hypercholesterolaemia, uncontrolled hypertension or diabetes, active liver disease or history of malignancy. Eligible patients were randomized (2:1) in permuted blocks to treatment with obicetrapib 10 mg or matching placebo, administered orally daily for 365 days. The primary endpoint was the per cent change in LDL-C from baseline to day 84. Secondary endpoints included the change in LDL-C from baseline to day 365, changes in other lipid and lipoprotein parameters and the per cent of patients achieving various LDL-C goals.

### Biochemical analysis

Analysis of cholesterol and triglycerides was by enzymatic methods on a Beckman Coulter AU Series automated analyzer. HDL-C was performed by precipitation with 50 kDa dextran sulfate with magnesium ions, followed by analysis of the supernatant for cholesterol by enzymatic methods. LDL-C was measured by preparative ultracentrifugation and subsequent quantitative measurement of cholesterol in HDL and LDL fractions by enzymatic methods. LDL-C was also determined by the Friedewald and Martin-Hopkins formulae. For the analyses the Martin-Hopkins formulae was used, unless the value was 50 mg/dL or less or triglycerides were at least 400 mg/dL, in which case the LDL-C determined by preparative ultracentrifugation was employed. Lp(a) was measured by a immunoturbidometric assay analysed on a Roche c501/c502 analyzer, performed using a molar method with calibration standardized against the IFCC reference material SRM2B for nmol/L. Apolipoprotein B (apoB) was by nephelometry on a Siemens BNII analyzer. Non-Lp(a) apoB was determined by subtracting Lp(a) from apoB, after converting to molar concentration.

### Statistical analysis

Analyses were performed on the prespecified modified on-treatment population from each study, consisting of all randomized participants who have both baseline and week 12 Lp(a) and LDL-C assessments; additionally, participants in the obicetrapib group must have week 12 plasma obicetrapib concentration no less than three standard deviations below the mean concentration in that treatment group. Categorical variables are summarized by frequency and percentage of patients while continuous variables are summarized by mean and standard deviation or median and interquartile range. Comparisons of changes from baseline to week 12 in lipids and lipoproteins between treatment groups are by two sample Hodges-Lehmann estimates of the midpoint of corresponding 95% confidence intervals (CIs) for location shift and Wilcoxon rank sum test *P*-values; results reflect stratification by baseline Lp(a) category (<50 nmol/L, 50 to <150 nmol/L, or ≥150 nmol/L) or tertile of each lipid/lipoprotein and study. Significance of interactions between categories of baseline Lp(a) and treatment for the median absolute and per cent change in Lp(a) were estimated in quantile regression models with change as the outcome, terms for Lp(a) category, treatment and their interaction, and study as an additional covariate. Percentages, 95% CIs, and *P*-values for proportions of participants achieving Lp(a) and LDL-C goals at baseline and week 12 are from logistic regression models with no adjustment for covariates. Within the obicetrapib group, Spearman correlations summarize relationships between change in Lp(a) and LDL-C. Relationships between baseline Lp(a) and change in Lp(a) are depicted by LOWESS curves with 10 datapoints in the smoothing windows to reduce the influence of individual outliers (e.g. large percentage increases among participants with low baseline concentrations).

## Results

### Baseline characteristics

Of the 2361 patients in the modified intention-to-treat population, 2356 (99.8%) had baseline and week 12 Lp(a) assessments and were included in the analyses. Clinical characteristics and baseline use of lipid-lowering medications are summarized in *Table 1*. No differences were observed between the treatment groups, apart from less individuals identifying as white race in the obicetrapib group. Mean age was 64 years, 36.2% were female and medical history included ASCVD in 82.3%, HeFH in 27.1%, diabetes in 33.1%, and hypertension in 76.6%. Lipid-lowering medication use included statins in 91.3%, high-intensity statins in 68.3%, ezetimibe in 29.2% and PCSK9 inhibitors in 5.6%.

**Table 1:** Clinical characteristics and medication use at baseline

Variable	Total (*n* = 2356)	Placebo (*n* = 920)	Obicetrapib (*n* = 1436)
Age, years	64 (11)	64 (10)	64 (11)
Females, *n* (%)	854 (36.2)	329 (35.8)	525 (36.6)
White race, *n* (%)	1754 (74.4)	724 (78.7)	1030 (71.7)
Body mass index, kg/m^2^	29.4 (5.5)	29.7 (5.7)	29.3 (5.4)
History of ASCVD, *n* (%)	1939 (82.3)	757 (82.3)	1182 (82.3)
Heterozygous FH, *n* (%)	638 (27.1)	250 (27.2)	388 (27.0)
Diabetes, *n* (%)	839 (33.1)	344 (37.4)	495 (34.4)
Hypertension, *n* (%)	1805 (76.6)	715 (77.7)	1090 (75.9)
Medication use			
Statins, *n* (%)	2150 (91.3)	842 (91.5)	1308 (91.1)
High intensity statins, *n* (%)	1608 (68.3)	632 (68.7)	976 (68.0)
Ezetimibe, *n* (%)	687 (29.2)	265 (28.8)	422 (29.4)
PCSK9 inhibitor, *n* (%)	132 (5.6)	59 (6.4)	73 (5.1)

Continuous variables are presented as mean (standard deviation) and categorical variables as number (percentage).

ASCVD, atherosclerotic cardiovascular disease; FH, familial hypercholesterolemia; PCSK9, proprotein convertase subtilisin/kexin type 9.

### Changes in lipoprotein(a)

**Figure 1:**
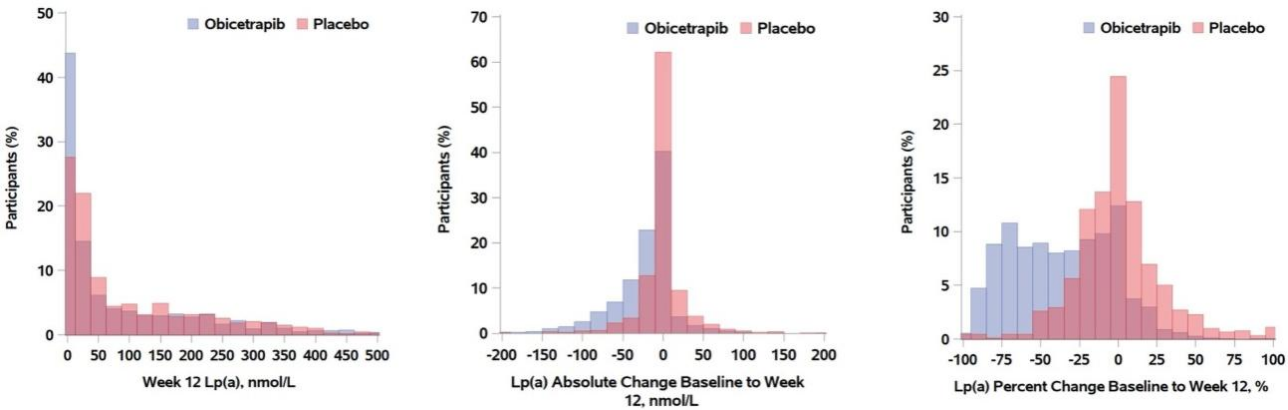
Distribution of on-treatment levels at week 12 (left panel) and both absolute (middle panel) and per cent (right panel) change from baseline in lipoprotein(a) [Lp(a)] in patients treated with obicetrapib or placebo

**Figure 2:**
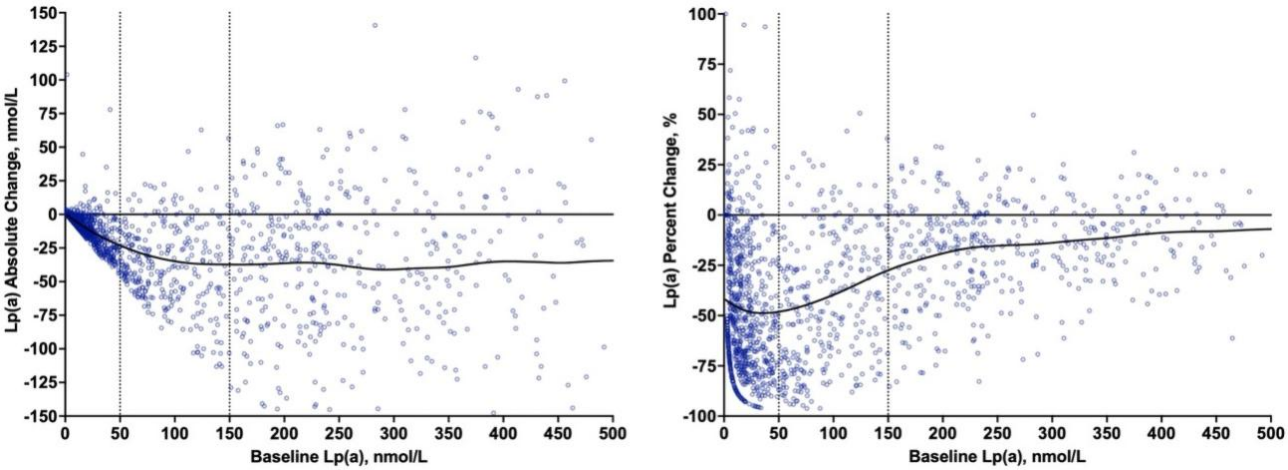
Scatterplot demonstrating absolute (left panel) and percentage (right panel) change in lipoprotein(a) [Lp(a)] according to baseline levels in the obicetrapib treatment group. LOWESS smoothing curves (solid lines) illustrate the relationship between baseline concentration and observed change

Lipoprotein(a) levels at baseline, week 12 and the serial change in the overall cohort and in patients with different ranges of baseline Lp(a) levels are summarized in *Table 2*, Supplementary data online, *Tables S1*-*S3* and *Figure 1*. Median baseline Lp(a) levels were 42.9 nmol/L, with 448 (19.0%) demonstrating levels between 50 and 150 nmol/L and 665 (28.2%) demonstrating levels at least 150 nmol/L. In the overall cohort, obicetrapib produced placebo-adjusted percentage (−37.3%) and absolute (−14.9 nmol/L) reductions in Lp(a) (*P* < .0001). There was significant heterogeneity in the obicetrapib treatment effect by categories of baseline Lp(a) for both absolute (*P*_interaction_ < .0001) and per cent (P_interaction_ < .0001) change. While a greater placebo-adjusted percentage reduction in Lp(a) with obicetrapib was observed in patients with baseline Lp(a) levels between 50 and 150 nmol/L (−43.3%, *P* < .0001) than levels at least 150 nmol/L (−12.6%, *P* < .0001), comparable placebo-adjusted absolute Lp(a) reductions with obicetrapib were observed at both strata (−36.3 nmol/L, *P* < .0001 and −32.3 nmol/L, *P* < .0001) (*Figure 2*). In contrast, the absolute and percentage placebo-adjusted reductions in LDL-C with obicetrapib were consistent in patients with different baseline Lp(a) levels (see Supplementary data online, *Table S1*). *Table 3* summarizes the correlation between changes in LDL-C, Lp(a) and apoB measures in the obicetrapib group. Modest, albeit statistically significant, associations were observed between both absolute and percentage changes in Lp(a) with either LDL-C or apoB. As expected, a stronger correlation was observed between changes in LDL-C and apoB.

**Table 2:** Lipoprotein(a) levels at baseline, week 12, and the serial changes in patients treated with placebo or obicetrapib

Overall cohort				
	Placebo (*n* = 920)	Obicetrapib (*n* = 1436)	Placebo-adjusted	*P*-value
Baseline (nmol/L)	38.6 (11.9, 161.7)	44.5 (12.6, 179.1)		
Week 12 (nmol/L)	38.4 (10.9, 156.2)	18.8 (4.7, 135.9)	−13.0 (−15.7, −10.4)	<.0001
Absolute change (nmol/L)	0 (−7.5, 5.0)	−11.4 (−34.9, −1.9)	−14.9 (−16.7, −13.1)	<.0001
Per cent change	0 (−15.2, 13.6)	−35.4 (−65.0, −8.5)	−37.3 (−40.0, −34.7)	<.0001

Baseline Lp(a) < 50 nmol/L				
	Placebo (*n* = 501)	Obicetrapib (*n* = 742)	Placebo-adjusted	*P*-value
Baseline (nmol/L)	13.5 (5.7, 24.8)	13.5 (5.3, 24.4)		
Week 12 (nmol/L)	12.5 (5.6, 24.7)	5.0 (2.8, 10.8)	−6.1 (−7.2, −4.9)	<.0001
Absolute change (nmol/L)	0 (−2.0, 2.2)	−5.0 (−13.4, −1.6)	−6.2 (−7.0, −5.3)	<.0001
Per cent change	0 (−18.1, 22.6)	−52.4 (−70.8, −18.0)	−50.9 (−54.8, −47.1)	<.0001

Baseline Lp(a) 50 to <150 nmol/L				
	Placebo (*n* = 170)	Obicetrapib (*n* = 278)	Placebo-adjusted	*P*-value
Baseline (nmol/L)	94.2 (67.3, 117.4)	89.2 (66.8, 116.9)		
Week 12 (nmol/L)	92.5 (62.5, 121.5)	44.8 (20.9, 83.7)	−38.7 (−46.0, −31.4)	<.0001
Absolute change (nmol/L)	−4.5 (−12.3, 8.8)	−39.5 (−57.2, −18.1)	−36.3 (−41.2, −31.4)	<.0001
Per cent change	−4.1 (−16.3, 10.0)	−46.3 (−71.1, −21.5)	−43.3 (−49.5, −37.1)	<.0001

Baseline Lp(a) ≥150 nmol/L				
	Placebo (*n* = 249)	Obicetrapib (*n* = 416)	Placebo-adjusted	*P*-value
Baseline (nmol/L)	249.6 (197.5, 341.0)	249.0 (200.3, 347.2)		
Week 12 (nmol/L)	251.6 (191.8, 331.8)	225.0 (161.3, 315.6)	−31.4 (−48.2, −14.6)	.0003
Absolute change (nmol/L)	−2.5 (−25.6, 20.7)	−31.8 (−77.8, 2.2)	−32.3 (−40.5, −24.1)	<.0001
Per cent change	−1.3 (−9.5, 8.8)	−13.3 (−27.7, .8)	−12.6 (−15.6, −9.6)	<.0001

Lp(a), lipoprotein(a).

Data are presented as median (interquartile range) for each treatment group and two sample Hodges-Lehmann median of differences 95% confidence interval midpoint for placebo-adjusted differences, reflecting stratification for Lp(a) category (<50 nmol/L, 50 to <150 nmol/L, or ≥150 nmol/L) and study. *P*-values are from Wilcoxon rank sum tests. *P*_interaction_ between treatment and baseline Lp(a) category (<50 nmol/L, 50 to <150 nmol/L, or ≥150 nmol/L) < .0001 and <.0001 for absolute and per cent change, respectively.

**Table 3:** Correlations between both absolute and percentage changes in lipid and lipoprotein parameters in the obicetrapib group

Parameters	Correlation *r* (95% CI)	*P*-value
Absolute change LDL-C and Lp(a)	.16 (.11, .21)	<.0001
Percent change LDL-C and Lp(a)	.31 (.26, .35)	<.0001
Absolute change apoB and LDL-C	.88 (.87, .89)	<.0001
Percent change apoB and LDL-C	.79 (.77, .81)	<.0001
Absolute change apoB and Lp(a)	.18 (.13, .23)	<.0001
Percent change apoB and Lp(a)	.38 (.33, .42)	<.0001
Absolute change non-Lp(a) apoB and Lp(a)	.09 (.04, .14)	=.0004
Percent change non-Lp(a) apoB and Lp(a)	.29 (.24, .33)	<.0001

CI, confidence interval; LDL-C, low-density lipoprotein cholesterol; apoB, apolipoprotein B; Lp(a), lipoprotein(a).

### Lipid and lipoprotein levels

Lipid and lipoprotein levels at different time points and their change from baseline to 12 weeks are summarized in *Tables 4* and Supplementary data online, *Tables S4*-*S6*. Median baseline levels included LDL-C 92 mg/dL, HDL-C 48 mg/dL, triglycerides 127 mg/dL, apolipoprotein B (apoB) 88 mg/dL and non-Lp(a) apoB 1501 nmol/L. Greater reductions from baseline were observed in the obicetrapib compared with the placebo group for LDL-C (−41.2 vs −2.7%; −37 vs −3 mg/dL, *P* < .0001), triglycerides (−7.4% vs 0%; −8 vs 0 mg/dL, *P* < .0001), apoB (−24.1 vs −1.1%; −21 vs −1 mg/dL, *P* < .0001) and non-Lp(a) apoB (−24.2 vs −1.2%; −35.4 vs −17 nmol/L, *P* < .0001). Greater increases in HDL-C were observed in the obicetrapib compared with the placebo group (+142.9% vs 0%; + 68.0 vs 0 mg/dL, *P* < .0001).

**Table 4:** Lipid and lipoprotein levels at baseline, week 12, and change in patients treated with placebo or obicetrapib

Parameter	Placebo (*n* = 920)	Obicetrapib (*n* = 1436)	Placebo-adjusted	*P*-value
Low-density lipoprotein cholesterol				
Baseline (mg/dL)	92 (75, 124)	92 (76, 120)		
Week 12 (mg/dL)	89 (71, 117)	55 (39, 79)	−35 (−38, −33)	<.0001
Absolute change (mg/dL)	−3 (−16, 9)	−37 (−58, −20)	−35 (−37, −33)	<.0001
Percent change	−2.7 (−16.1, 10.6)	−41.2 (−55.0, −22.7)	−37.0 (−38.9, −35.1)	<.0001
High-density lipoprotein cholesterol				
Baseline (mg/dL)	48 (39, 57)	48 (40, 58)		
Week 12 (mg/dL)	47 (38, 58)	116 (98, 134)	68 (67, 70)	<.0001
Absolute change (mg/dL)	0 (−5, 4)	68 (52, 81)	68 (67, 69)	<.0001
Percent change	0 (−9.8, 9.1)	142.9 (103.0, 178.2)	142.3 (139.1, 145.5)	<.0001
Triglycerides				
Baseline (mg/dL)	127 (91, 176)	122 (88, 166)		
Week 12 (mg/dL)	126 (92,177)	109 (85, 143)	−12 (−15, −9)	<.0001
Absolute change (mg/dL)	0 (−26, 25)	−8 (−36, 13)	−13 (−16, −9)	<.0001
Percent change	0 (−19.1, 23.8)	−7.4 (−25.6, 14.1)	−8.5 (−11.0, −6.1)	<.0001
Apolipoprotein B				
Baseline (mg/dL)	88 (74, 108)	87 (75, 105)		
Week 12 (mg/dL)	86 (71, 104)	66 (57, 80)	−19 (−21, −18)	<.0001
Absolute change (mg/dL)	−1 (−12, 8)	−21 (−35, −8)	−20 (−21, −18)	<.0001
Percent change	−1.1 (−12.4, 9.3)	−24.1 (−35.2, −10.3)	−21.3 (−22.7, −19.9)	<.0001
Non-Lp(a) apolipoprotein B				
Baseline (nmol/L)	1501 (1230, 1866)	1480 (1234, 1824)		
Week 12 (nmol/L)	1458 (1188, 1797)	1123 (944, 1359)	−323 (−349, −297)	<.0001
Absolute change (nmol/L)	−17 (−215, 143)	−354 (−611, −121)	−335 (−360, −311)	<.0001
Percent change	−1.2 (−13.2, 10.0)	−24.2 (−35.7, −9.9)	−21.5 (−23.0, −20.1)	<.0001

Data are presented as median (interquartile range) for each treatment group and two sample Hodges-Lehmann median of differences confidence interval midpoint (95% CI) for placebo adjusted differences, reflecting stratification for baseline tertile and study. *P*-values are from Wilcoxon rank sum tests.

### Combined achievement of LDL-C and Lp(a)

**Figure 3:**
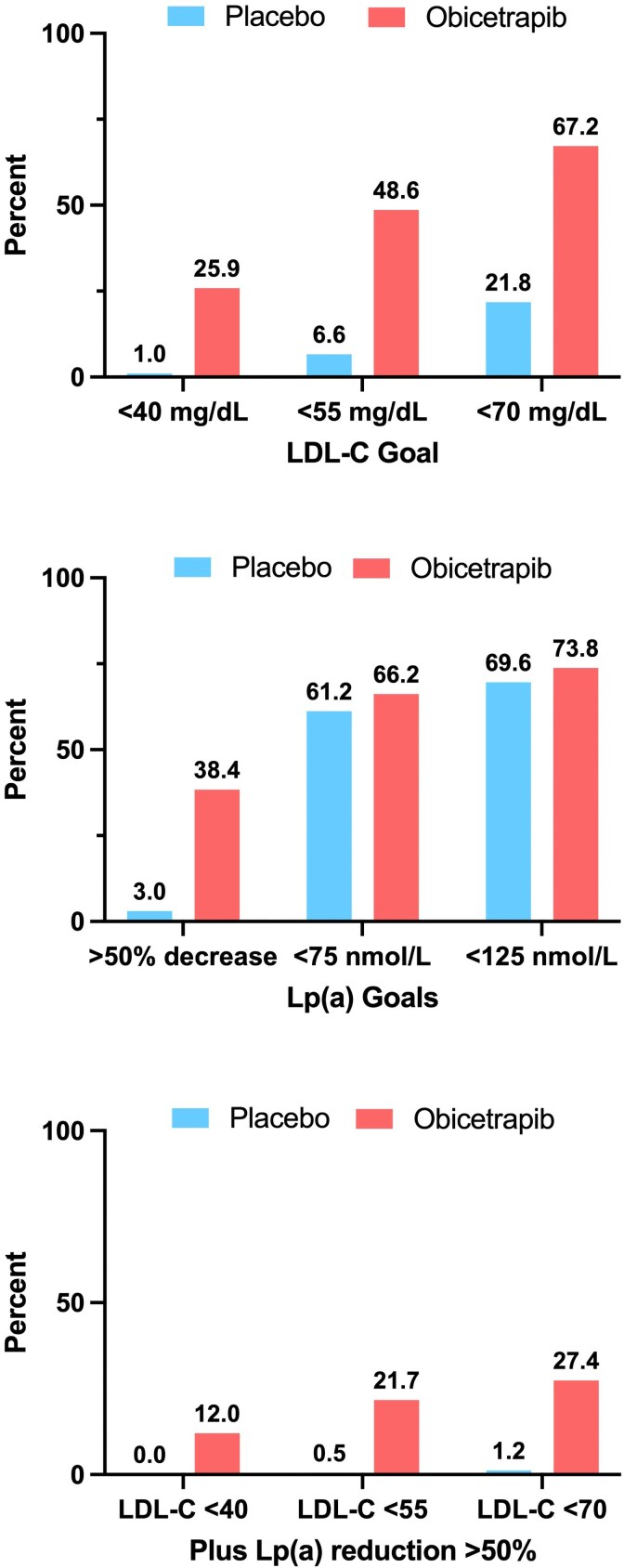
Percentage of patients treated with obicetrapib or placebo achieving treatment goals of low-density lipoprotein cholesterol (LDL-C), lipoprotein(a) [Lp(a)] and their combination

The percentage of patients achieving various degrees of change or achieved levels of LDL-C and Lp(a) during the study and their combination are summarized in *Table 5* and *Figure 3*. A greater percentage of obicetrapib-treated patients achieved LDL-C levels <40 mg/dL (25.9% vs 1.0%, *P* < .0001), 55 mg/dL (48.6% vs 6.6%, *P* < .0001) and 70 mg/dL (67.2% vs 21.8%, *P* < .0001). A greater percentage of obicetrapib treated patients achieved Lp(a) lowering >50% from baseline (38.4% vs 3.0%, *P* < .0001) or on-treatment levels <75 nmol/L (66.2% vs 61.2%, *P* = .01) and 125 nmol/L (73.8% vs 69.6%, *P* = .02). Patients treated with obicetrapib were more likely to achieve both a combination of Lp(a) lowering >50% and an on-treatment LDL-C <40 mg/dL (12.0% vs 0%, *P* < .0001), 55 mg/dL (21.7% vs .5%, *P* < .0001) and 70 mg/dL (27.4% vs 1.2%, *P* < .0001). A greater percentage of obicetrapib-treated patients also achieved the combination of the LDL-C goals and either a Lp(a) <75 or 125 nmol/L (*Structured Graphical Abstract*).

**Table 5:** Percentage of patients treated with obicetrapib or placebo on achieved treatment goals of LDL-C and Lp(a) and their combination

Goal	Timepoint	Obicetrapib Percent (95% CI)	Placebo Percent (95% CI)	Difference (95% CI)	*P*-value
	Achieved LDL-C				
LDL-C < 40 mg/dL	Baseline	.4 (.2, .9)	.4 (.2, 1.1)	0 (−.5, .5)	.96
	Week 12	25.9 (23.7, 28.3)	1.0 (.5, 1.9)	25.0 (22.6, 27.3)	<.0001
LDL-C < 55 mg/dL	Baseline	4.0 (3.1, 5.1)	3.9 (2.8, 5.4)	.1 (−1.5, 1.7)	.92
	Week 12	48.6 (46.1, 51.2)	6.6 (5.2, 8.4)	42.0 (39.0, 45.1)	<.0001
LDL-C < 70 mg/dL	Baseline	16.4 (14.5, 18.4)	16.2 (14.0, 18.8)	.1 (−2.9, 3.2)	.94
	Week 12	67.2 (64.7, 69.5)	21.8 (19.2, 24.5)	45.4 (41.8, 49.0)	<.0001
	Achieved Lp(a)				
Lp(a) > 50% decrease	Baseline	N/A	N/A		N/A
	Week 12	38.4 (36.0, 41.0)	3.0 (2.1, 4.4)	35.4 (32.7, 38.2)	<.0001
Lp(a) < 75 nmol/L	Baseline	58.6 (56.0, 61.1)	60.2 (57.0, 63.3)	−1.7 (−5.7, 2.4)	.43
	Week 12	66.2 (63.7, 68.6)	61.2 (58.0, 64.3)	5.0 (1.0 9.0)	.01
Lp(a) < 125 nmol/L	Baseline	67.6 (65.1, 69.9)	70.1 (67.1, 73.0)	−2.6 (−6.4, 1.3)	.19
	Week 12	73.8 (71.5, 76.0)	69.6 (66.5, 72.5)	4.3 (.5, 8.0)	.02
	Combined Achieved LDL-C and Lp(a)				
LDL-C < 40 mg/dL and Lp(a) > 50% decrease	Baseline	N/A	N/A		N/A
	Week 12	12.0 (10.4, 13.7)	0 (0, .4)	12.0 (10.3, 13.6)	<.0001
LDL-C < 40 mg/dL and Lp(a) < 75 nmol/L	Baseline	.2 (.1, .6)	.3 (.1, 1.0)	−.1 (−.6, .3)	.59
	Week 12	19.4 (17.4, 21.5)	.8 (.4, 1.6)	18.6 (16.5, 20.7)	<.0001
LDL-C < 40 mg/dL and Lp(a) < 125 nmol/L	Baseline	.2 (.1, .6)	.3 (.1, 1.0)	−.1 (−.6, .3)	.59
	Week 12	21.5 (19.5, 23.8)	.9 (.4, 1.7)	20.7 (18.5, 22.9)	<.0001
LDL-C < 55 mg/dL and Lp(a) > 50% decrease	Baseline	N/A	N/A		N/A
	Week 12	21.7 (19.6, 23.9)	.5 (.2, 1.3)	21.1 (19.0, 23.3)	<.0001
LDL-C < 55 mg/dL and Lp(a) < 75 nmol/L	Baseline	2.5 (1.8, 3.5)	2.4 (1.6, 3.6)	.1 (−1.1, 1.4)	.85
	Week 12	35.2 (32.8, 37.8)	4.5 (3.3, 6.0)	30.8 (28.0, 33.6)	<.0001
LDL-C < 55 mg/dL and Lp(a) < 125 nmol/L	Baseline	3.0 (2.2, 4.0)	2.5 (1.7, 3.7)	.5 (−.8, 1.9)	.47
	Week 12	39.0 (36.5, 41.6)	5.0 (3.8, 6.6)	34.0 (31.1, 36.9)	<.0001
LDL-C < 70 mg/dL and Lp(a) > 50% decrease	Baseline	N/A	N/A		N/A
	Week 12	27.4 (25.2, 29.8)	1.2 (.7, 2.1)	26.2 (23.8, 28.6)	<.0001
LDL-C < 70 mg/dL and Lp(a) < 75 nmol/L	Baseline	9.4 (8.0, 11.0)	8.7 (7.0, 10.7)	.7 (−1.7, 3.0)	.58
	Week 12	45.9 (43.4, 48.5)	14.2 (12.1, 16.7)	31.7 (28.3, 35.1)	<.0001
LDL-C < 70 mg/dL and Lp(a) < 125 nmol/L	Baseline	10.6 (9.1, 12.3)	10.4 (8.6, 12.6)	.1 (−2.4, 2.6)	.92
	Week 12	51.0 (48.4, 53.6)	15.8 (13.6, 18.3)	35.2 (31.7, 38.7)	<.0001

*P*-values are from Wald tests.

CI, confidence interval; LDL-C, low-density lipoprotein cholesterol; Lp(a), lipoprotein(a); N/A, not available.

## Discussion

This pooled analysis of two phase 3 clinical trials that investigated the impact of obicetrapib on lipid levels with 12 months of treatment provided the opportunity to more extensively characterize its effects on Lp(a) levels. In addition to lowering levels of LDL-C by 37% and apoB by 21%, treatment with obicetrapib produced median placebo-adjusted reductions in Lp(a) by 37.3% and 14.9 nmol/L. In patients with baseline Lp(a) levels between at least 50 and 150 nmol/L, obicetrapib lowered Lp(a) by 43.3% and 36.3 nmol/L, while patients with higher baseline Lp(a) levels demonstrated lowering with obicetrapib by 12.6% and 32.3 nmol/L. Obicetrapib enabled more patients to achieve concurrent lowering of both LDL-C and Lp(a) to recommended targets.

The greater focus on the clinical consequences of high Lp(a) levels raises important questions on what role it will play in approaches to the prevention of cardiovascular disease. Elevated Lp(a) levels identify residual risk in statin-treated patients. Numerous treatment guidelines recommend Lp(a) testing due to its ability to reclassify risk in more than 40% of individuals. However, the poor uptake of Lp(a) testing is driven by the lack of subsidization of testing in most countries, limited knowledge of the importance of Lp(a) and considerable perception in clinical practice that there is nothing that can currently be done for patients with high Lp(a) levels. Several lines of observational evidence suggest that the elevated risk associated with a high Lp(a) can be modified, including adopting more healthy lifestyle habits, intensification of lowering of blood pressure and cholesterol and use of aspirin and PCSK9 inhibitors.^13^ However, there is an interest in the development of novel therapeutics that can specifically address Lp(a).

A number of programmes have developed novel therapeutic agents with the potential to selectively lower Lp(a) levels. This has primarily focused on the development of injectable agents that target RNA regulating apolipoprotein(a) [apo(a)] synthesis. Pelacarsen is an antisense oligonucleotide that lowers Lp(a) by up to 80%^14,15^ and three short interfering RNA agents have been demonstrated to lower Lp(a) by more than 95%.^16–20^ Many of these agents are currently being investigated in large cardiovascular outcome trials of patients at high risk of cardiovascular events and with very high Lp(a) levels.^21–23^ Muvalaplin is a small molecule that inhibits the binding of apo(a) and apoB in the hepatocyte and disrupts the assembly of Lp(a), lowering circulating levels by up to 85%.^24^ These agents will need to demonstrate effective lowering of cardiovascular events and good tolerability to present an additional clinical tool for the reduction of cardiovascular risk.

In the event that these agents do prove to reduce cardiovascular risk, interest will focus on how to rapidly integrate them into clinical practice. The need for injectable administration and high cost will likely constrain their use to those with only the highest Lp(a) levels. While an oral agent may overcome some of these barriers, muvalaplin has not yet progressed to a cardiovascular outcome trial. The very high Lp(a) levels required for inclusion in the outcomes trials, being more than 70 mg/dL (∼175 nmol/L) for pelacarsen, 175 nmol/L for lepodisiran and 200 nmol/L for olpasiran, will leave many patients with high Lp(a) levels unable to access therapies. It is well recognized that cardiovascular risk increases at Lp(a) levels >50 nmol/L and guidelines advocate the importance of targeting patients with levels >125 nmol/L. Given the skewed distribution of Lp(a) in the population, there is likely to be a large burden of patients with high Lp(a) levels who will be left requiring effective therapeutic approaches in the clinic.

The findings contribute to our understanding of the effects of CETP inhibition with obicetrapib and their potential contribution to the prevention of cardiovascular disease. *Post hoc* review of previous development programmes and insights from genomic analyses have informed the transition of focus on CETP inhibitors from raising HDL-C to lowering levels of atherogenic lipids and lipoproteins. This underscored the development of obicetrapib, which effectively lowers LDL-C and apoB, and is currently being evaluated in clinical trials of patients with suboptimally controlled LDL-C levels. The finding that obicetrapib also lowers Lp(a), independently of its effects on LDL-C, provides another biochemical effect with the potential to reduce cardiovascular risk. The combination of lowering both LDL-C and Lp(a) may be an attractive therapeutic option in the management of patients at high risk of cardiovascular events. The ability to demonstrate a reduction in cardiovascular events will be essential to progress obicetrapib to the prevention clinic.

The pattern of Lp(a) reduction observed with obicetrapib can be understood in the context of findings from large-scale PCSK9 inhibitor trials. In the ODYSSEY OUTCOMES trial with alirocumab and the FOURIER trial with evolocumab, totalling over 45 000 patients with ASCVD, a consistent pattern was demonstrated wherein Lp(a) reductions were lower among those with higher baseline levels.^5,6^ In ODYSSEY OUTCOMES, alirocumab demonstrated greater Lp(a) reductions in patients with baseline levels below 125 nmol/L compared to those with levels above 125 nmol/L.^6^ Our observations with obicetrapib are similar. Both PCSK9 inhibitors and CETP inhibitors appear to deviate from Wilder's Law of initial value that typically governs dose-response relationships for cholesterol and blood pressure medications.

The mechanistic basis for these patterns relates to the unique biology of Lp(a) particle metabolism. Small isoform Lp(a) particles are characterized by high synthesis rates. Kinetic studies have demonstrated that isoforms with fewer kringle IV type 2 repeats exhibit higher production rates compared to larger isoforms. These smaller isoforms lead to elevated Lp(a) levels due to more efficient hepatic secretion and less intracellular degradation.^25^ Such high production rates are countered most effectively by interfering with intracellular synthesis through RNA inhibition, and the latest generation of apo(a) antisense oligonucleotides and siRNA inhibitors demonstrate potent and sustained Lp(a) lowering.^14–19^ The two other classes of lipid-modifying drugs, PCSK9 monoclonal antibodies and CETP inhibitors, are unlikely to directly affect intrahepatic apo(a) synthesis rates, since both drugs exert their primary effects through extracellular mechanisms. The clinical significance of these pharmacological patterns will be established through ongoing cardiovascular outcome trials.

A number of limitations should be highlighted. This analysis pooled data from two trials of high cardiovascular risk patients who were treated with obicetrapib or placebo for 365 days, although they demonstrated heterogeneity in terms of background clinical history and qualifying lipid levels. Patients were not required to have elevated Lp(a) levels, accordingly the majority had a Lp(a) concentration within the normal range. Additional studies will specifically investigate the impact of obicetrapib in cohorts with high Lp(a) levels. The relationship between apo(a) isoform size and obicetrapib’s Lp(a)-lowering effects has not been characterized, and future investigations examining isoform-specific responses may provide additional insights into interindividual variability in treatment response. The longer-term effects of obicetrapib on Lp(a) will need to be determined in ongoing studies. Other Lp(a) lowering agents have been demonstrated to reduce levels of oxidized phospholipids, a factor implicated in the atherogenicity of Lp(a), this remains to be investigated in obicetrapib treated patients. While a more modest degree of Lp(a) lowering has been observed to independently associate with the reduction in cardiovascular events with PCSK9 inhibitors, we await definitive evidence that specific lowering of Lp(a) leads to a reduction in cardiovascular risk. Furthermore, the degree of Lp(a) lowering required to produce a clinically meaningful reduction in cardiovascular risk, reflecting at least a 15% reduction in major adverse cardiovascular events, remains to be established.

In combination with its effects on LDL-C and apoB, administration of obicetrapib resulted in reductions in circulating concentrations of Lp(a), further establishing the ability of obicetrapib to favourably modulate levels of lipid and lipoprotein factors implicated in the causality of ASCVD.

## Supplementary Material

ehag399_Supplementary_Data

## Declarations

### Data Availability

No data will be made available at this point in time.

### Pre-registered clinical trial number

BROADWAY: NCT05142722

BROOKLYN: NCT05425745
