# Supplementary material for: Obicetrapib and lipoprotein(a) levels in patients at high cardiovascular risk: a pooled analysis of trials
Source: Eur Heart J. 2026 May 25;47(26):3404–14. doi: 10.1093/eurheartj/ehag399 (PMC13337231; doi:10.1093/eurheartj/ehag399)
Supplement: ehag399_Supplementary_Data [file ehag399_supplementary_data.docx]

**Table S1. Sensitivity Analysis of Lipoprotein(a)**

| **Overall Cohort** | | | | |
| --- | --- | --- | --- | --- |
|  | **Placebo**  **(N=920)** | **Obicetrapib**  **(N=1855)** | **Placebo-Adjusted** | **P Value** |
| Baseline (nmol/L) | 38.6 (11.9, 161.7) | 41.0 (11.6, 168.2) |  |  |
| Week 12 (nmol/L) | 38.4 (10.9, 156.2) | 18.2 (4.6, 129.8) | -8.0 (-10.6, -5.3) | <0.0001 |
| Absolute Change (nmol/L) | 0 (-7.5, 5.0) | -9.8 (-33.7, -1.5) | -11.9 (-13.5, -10.2) | <0.0001 |
| Percent Change | 0 (-15.2, 13.6) | -32.5 (-63.0, -5.1) | -34.9 (-37.9, -32.0) | <0.0001 |
| **Baseline Lp(a) <50 nmol/L** | | | | |
|  | **Placebo**  **(N=501)** | **Obicetrapib**  **(N=992)** | **Placebo-Adjusted** | **P Value** |
| Baseline (nmol/L) | 13.5 (5.7, 24.8) | 12.5 (5.2, 24.2) |  |  |
| Week 12 (nmol/L) | 12.5 (5.6, 24.7) | 5.2 (2.8, 11.2) | -5.8 (-6.9, -4.7) | <0.0001 |
| Absolute Change (nmol/L) | 0 (-2.0, 2.2) | -4.3 (-12.3, -1.0) | -5.4 (-6.2, -4.6) | <0.0001 |
| Percent Change | 0 (-18.1, 22.6) | -49.4 (-69.4, -10.0) | -47.4 (-51.5, -43.4) | <0.0001 |
| **Baseline Lp(a) 50 to <150 nmol/L** | | | | |
|  | **Placebo**  **(N=170)** | **Obicetrapib**  **(N=344)** | **Placebo-Adjusted** | **P Value** |
| Baseline (nmol/L) | 94.2 (67.3, 117.4) | 90.3 (67.0, 120.4) |  |  |
| Week 12 (nmol/L) | 92.5 (62.5, 121.5) | 47.7 (26.3, 85.7) | -36.9 (-43.9, -29.8) | <0.0001 |
| Absolute Change (nmol/L) | -4.5 (-12.3, 8.8) | -37.6 (-56.1, -13.3) | -34.6 (-39.4, -29.7) | <0.0001 |
| Percent Change | -4.1 (-16.3, 10.0) | -42.1 (-69.1, -16.0) | -40.7 (-46.9, -34.5) | <0.0001 |
| **Baseline Lp(a) ≥150 nmol/L** | | | | |
|  | **Placebo**  **(N=249)** | **Obicetrapib**  **(N=519)** | **Placebo-Adjusted** | **P Value** |
| Baseline (nmol/L) | 249.6 (197.5, 341.0) | 243.0 (198.0, 341.6) |  |  |
| Week 12 (nmol/L) | 251.6 (191.8, 331.8) | 217.4 (157.6, 313.6) | -37.2 (-53.3, -21.0) | <0.0001 |
| Absolute Change (nmol/L) | -2.5 (-25.6, 20.7) | -33.3 (-76.2, 1.1) | -33.1 (-40.9, -25.2) | <0.0001 |
| Percent Change | -1.3 (-9.5, 8.8) | -13.3 (-28.1, 0.4) | -13.0 (-15.9, -10.3) | <0.0001 |

Table S1. Sensitvity analyses of lipoprotein(a) levels at baseline, Week 12, and change in patients treated with placebo or obicetrapib presented as median (interquartile range) for each treatment group and Hodges-Lehmann median of differences confidence interval midpoint (95% CI) for placebo adjusted differences. Results reflect the intent-to-treat population from each study, consisting of all of all randomized participants who have both baseline and week 12 Lp(a) and LDL-C assessments. P-values are from Wilcoxon tests. P_interaction_ between treatment and baseline Lp(a) category (<50 nmol/L, 50 to <150 nmol/L, or $\geq$150 nmol/L) <0.0001 and <0.00001 for absolute and percent change, respectively.

**Table S2. Lipoprotein(a) in BROADWAY**

| **Overall Cohort** | | | | |
| --- | --- | --- | --- | --- |
|  | **Placebo**  **(N=805)** | **Obicetrapib**  **(N=1236)** | **Placebo-Adjusted** | **P Value** |
| Baseline (nmol/L) | 38.7 (11.8, 157.7) | 44.3 (12.6, 179.9) |  |  |
| Week 12 (nmol/L) | 38.4 (10.6, 151.3) | 18.8 (4.7, 137.6) | -6.9 (-9.6, -4.1) | <0.0001 |
| Absolute Change (nmol/L) | -0.2 (-7.6, 4.5) | -11.1 (-34.8, -1.8) | -12.8 (-14.7, -10.8) | <0.0001 |
| Percent Change | -0.9 (-15.7, 13.0) | -35.4 (-64.1, -8.2) | -36.3 (-39.5, -33.0) | <0.0001 |
| **Baseline Lp(a) <50 nmol/L** | | | | |
|  | **Placebo**  **(N=441)** | **Obicetrapib**  **(N=640)** | **Placebo-Adjusted** | **P Value** |
| Baseline (nmol/L) | 13.4 (5.6, 25.7) | 13.3 (5.3, 24.2) |  |  |
| Week 12 (nmol/L) | 12.4 (5.5, 24.7) | 5.0 (1.4, 10.8) | -6.0 (-7.2, -4.8) | <0.0001 |
| Absolute Change (nmol/L) | 0 (-2.1, 2.0) | -4.9 (-13.4, -1.6) | -5.8 (-6.7, -4.9) | <0.0001 |
| Percent Change | 0 (-19.1, 20.4) | -52.5 (-71.4, -18.2) | -49.8 (-54.0, -45.7) | <0.0001 |
| **Baseline Lp(a) 50 to <150 nmol/L** | | | | |
|  | **Placebo**  **(N=154)** | **Obicetrapib**  **(N=233)** | **Placebo-Adjusted** | **P Value** |
| Baseline (nmol/L) | 94.0 (67.2, 117.3) | 90.3 (67.5, 118.4) |  |  |
| Week 12 (nmol/L) | 92.5 (62.5, 120.1) | 46.0 (22.9, 85.8) | -38.0 (-45.7, -30.3) | <0.0001 |
| Absolute Change (nmol/L) | -4.6 (-12.6, 7.8) | -38.8 (-56.6, -17.7) | -35.8 (-40.9, -30.7) | <0.0001 |
| Percent Change | -4.2 (-16.3, 9.6) | -44.8 (-70.2, -21.0) | -42.8 (-49.5, -36.1) | <0.0001 |
| **Baseline Lp(a) ≥150 nmol/L** | | | | |
|  | **Placebo**  **(N=210)** | **Obicetrapib**  **(N=363)** | **Placebo-Adjusted** | **P Value** |
| Baseline (nmol/L) | 249.7 (199.4, 341.9) | 248.7 (198.6, 345.5) |  |  |
| Week 12 (nmol/L) | 255.4 (192.3, 331.8) | 224.2 (158.8, 315.3) | -34.0 (-52.0, -16.0) | 0.0003 |
| Absolute Change (nmol/L) | -3.6 (-25.6, 18.6) | -31.8 (-78.3, 2.5) | -31.7 (-40.7, -22.7) | <0.0001 |
| Percent Change | -1.3 (-9.4, 8.1) | -13.5 (-28.1, 1.1) | -12.6 (-15.9, -9.3) | <0.0001 |

Table S2. Lipoprotein(a) levels at baseline, Week 12, and change in patients treated with placebo or obicetrapib in the BROADWAY study presented as median (interquartile range) for each treatment group and Hodges-Lehmann median of differences confidence interval midpoint (95% CI) for placebo adjusted differences. Results reflect the intent-to-treat population from each study, consisting of all of all randomized participants who have both baseline and week 12 Lp(a) and LDL-C assessments. P-values are from Wilcoxon tests. P_interaction_ between treatment and baseline Lp(a) category (<50 nmol/L, 50 to <150 nmol/L, or $\geq$150 nmol/L) <0.0001 and <0.00001 for absolute and percent change, respectively.

**Table S3. Lipoprotein(a) in BROOKLYN**

| **Overall Cohort** | | | | |
| --- | --- | --- | --- | --- |
|  | **Placebo**  **(N=115)** | **Obicetrapib**  **(N=200)** | **Placebo-Adjusted** | **P Value** |
| Baseline (nmol/L) | 35.0 (13.3, 185.8) | 45.8 (15.4, 165.5) |  |  |
| Week 12 (nmol/L) | 39.9 (12.5, 191.8) | 18.6 (5.2, 94.6) | -12.9 (-21.9, -3.9) | 0.0007 |
| Absolute Change (nmol/L) | 0.7 (-6.9, 7.5) | -12.9 (-36.3, -2.5) | -18.1 (-24.1, -12.0) | <0.0001 |
| Percent Change | 3.7 (-9.3, 22.2) | -35.9 (-66.5, -10.3) | -44.9 (-53.1, -36.7) | <0.0001 |
| **Baseline Lp(a) <50 nmol/L** | | | | |
|  | **Placebo**  **(N=60)** | **Obicetrapib**  **(N=102)** | **Placebo-Adjusted** | **P Value** |
| Baseline (nmol/L) | 14.2 (6.3, 21.8) | 15.4 (5.6, 26.8) |  |  |
| Week 12 (nmol/L) | 15.4 (6.7, 25.1) | 5.6 (3.2, 11.2) | -7.2 (-10.5, -3.8) | <0.0001 |
| Absolute Change (nmol/L) | 1.0 (-0.3, 4.0) | -5.7 (-13.4, -1.6) | -8.1 (-10.4, -5.7) | <0.0001 |
| Percent Change | 7.8 (-4.6, 32.9) | -50.4 (-68.9, -16.1) | -58.1 (-68.8, -47.4) | <0.0001 |
| **Baseline Lp(a) 50 to <150 nmol/L** | | | | |
|  | **Placebo**  **(N=16)** | **Obicetrapib**  **(N=45)** | **Placebo-Adjusted** | **P Value** |
| Baseline (nmol/L) | 100.2 (74.5, 123.4) | 79.9 (60.5, 102.7) |  |  |
| Week 12 (nmol/L) | 96.7 (59.1, 143.2) | 38.2 (19.1, 59.1) | -55.8 (-82.8, -28.7) | 0.0001 |
| Absolute Change (nmol/L) | -2.7 (-10.8, 15.9) | -44.7 (-61.5, -22.3) | -43.5 (-60.4, -26.6) | <0.0001 |
| Percent Change | -2.4 (-16.1, 15.2) | -53.2 (-77.9, -28.6) | -52.2 (-70.6, -33.7) | <0.0001 |
| **Baseline Lp(a) ≥150 nmol/L** | | | | |
|  | **Placebo**  **(N=39)** | **Obicetrapib**  **(N=53)** | **Placebo-Adjusted** | **P Value** |
| Baseline (nmol/L) | 247.7 (182.9, 340.4) | 256.0 (217.1, 349.6) |  |  |
| Week 12 (nmol/L) | 238.1 (188.4, 355.8) | 237.5 (167.8, 319.2) | -18.5 (-68.8, 31.9) | 0.59 |
| Absolute Change (nmol/L) | 0.4 (-29.6, 34.0) | -31.3 (-69.8, -4.6) | -37.3 (-58.6, -16.0) | 0.0006 |
| Percent Change | 0.3 (-12.8, 10.9) | -10.4 (-20.9, -1.0) | -13.3 (-21.2, -5.3) | 0.0009 |

Table S3. Lipoprotein(a) levels at baseline, Week 12, and change in patients treated with placebo or obicetrapib in the BROOKLYN study presented as median (interquartile range) for each treatment group and Hodges-Lehmann median of differences confidence interval midpoint (95% CI) for placebo adjusted differences. Results reflect the intent-to-treat population from each study, consisting of all of all randomized participants who have both baseline and week 12 Lp(a) and LDL-C assessments. P-values are from Wilcoxon tests. P_interaction_ between treatment and baseline Lp(a) category (<50 nmol/L, 50 to <150 nmol/L, or $\geq$150 nmol/L) <0.0001 and <0.00001 for absolute and percent change, respectively.

**Table S4. Changes in LDL-C Overall and by Categories of Baseline Lp(a)**

| **Overall Cohort** | | | | |
| --- | --- | --- | --- | --- |
|  | **Placebo**  **(N=920)** | **Obicetrapib**  **(N=1436)** | **Placebo-Adjusted** | **P Value** |
| Baseline (mg/dL) | 92 (75, 124) | 92 (76, 120) |  |  |
| Week 12 (mg/dL) | 89 (71, 117) | 55 (39, 79) | -34 (-36, -31) | <0.0001 |
| Absolute Change mg/dL) | -3 (-16, 9) | -37 (-58, -20) | -34 (-36, -32) | <0.0001 |
| Percent Change | -2.7 (-16.1, 10.6) | -41.2 (-55.0, -22.7) | -37.1 (-39.0, -35.1) | <0.0001 |
| **Baseline Lp(a) <50 nmol/L** | | | | |
|  | **Placebo**  **(N=501)** | **Obicetrapib**  **(N=742)** | **Placebo-Adjusted** | **P Value** |
| Baseline (mg/dL) | 94 (75, 127) | 92 (76, 122) |  |  |
| Week 12 (mg/dL) | 90 (70, 120) | 52 (36, 75) | -38 (-41, -34) | <0.0001 |
| Absolute Change (mg/dL) | -3 (-17, 8) | -40 (-63, -22) | -37 (-40, -34) | <0.0001 |
| Percent Change | -3.2 (-17.6, 9.5) | -45.8 (-59.3, -26.0) | -39.5 (-42.2, -36.8) | <0.0001 |
| **Baseline Lp(a) 50 to <150 nmol/L** | | | | |
|  | **Placebo**  **(N=170)** | **Obicetrapib**  **(N=278)** | **Placebo-Adjusted** | **P Value** |
| Baseline (mg/dL) | 93 (77, 127) | 97 (78, 123) |  |  |
| Week 12 (mg/dL) | 89 (72, 119) | 57 (43, 84) | -32 (-37, -26) | <0.0001 |
| Absolute Change (mg/dL) | -4 (-18, 10) | -39 (-60, -21) | -35 (-40, -30) | <0.0001 |
| Percent Change | -4.0 (-17.5, 10.0) | -41.8 (-54.8, -24.6) | -36.6 (-41.2, -32.1) | <0.0001 |
| **Baseline Lp(a) ≥150 nmol/L** | | | | |
|  | **Placebo**  **(N=249)** | **Obicetrapib**  **(N=416)** | **Placebo-Adjusted** | **P Value** |
| Baseline (mg/dL) | 88 (73, 108) | 91 (75, 115) |  |  |
| Week 12 (mg/dL) | 87 (72, 106) | 61 (46, 83) | -27 (-31, -23) | <0.0001 |
| Absolute Change mg/dL) | 0 (-11, 12) | -30 (-46, -16) | -31 (-34, -27) | <0.0001 |
| Percent Change | 0 (-11.6, 12.8) | -35.2 (-47.5, -17.8) | -33.5 (-36.8, -30.2) | <0.0001 |

Table S4. Low-density lipoprotein cholesterol (LDL-C) levels at baseline, Week 12, and change in patients treated with placebo or obicetrapib in the overall cohort and in patients at different baseline levels of lipoprotein(a) [Lp(a)] presented as median (interquartile range) for each treatment group and Hodges-Lehmann median of differences confidence interval midpoint (95% CI) for placebo adjusted differences. P-values are from Wilcoxon tests.

**Table S5. Lipids and Lipoproteins in BROADWAY**

| **Parameter** | | **Placebo**  **(N=805)** | **Obicetrapib**  **(N=1236)** | **Placebo Adjusted** | **P Value** |
| --- | --- | --- | --- | --- | --- |
| Low-density lipoprotein cholesterol | |  |  |  |  |
|  | Baseline (mg/dL) | 90 (74, 118) | 90 (75, 116) |  |  |
|  | Week 12 (mg/dL) | 87 (70, 113) | 54 (38, 76) | -33 (-35, -30) | <0.0001 |
|  | Absolute change (mg/dL) | -3 (-17, 8) | -36 (-56, -20) | -33 (-35, -30) | <0.0001 |
|  | Percent change | -3.7 (-16.7, 10.4) | -41.4 (-55.3, -22.7) | -36.6 (-38.7, -34.4) | <0.0001 |
| High-density lipoprotein cholesterol | |  |  |  |  |
|  | Baseline (mg/dL) | 48 (39, 57) | 48 (39, 57) |  |  |
|  | Week 12 (mg/dL) | 47 (38, 57) | 116 (97, 134) | 67 (65, 69) | <0.0001 |
|  | Absolute change (mg/dL) | 0 (-5, 4) | 68 (52, 81) | 69 (67, 70) | <0.0001 |
|  | Percent change | 0 (-10.0, 8.9) | 143.4 (103.9, 181.3) | 142.0 (138.3, 145.8) | <0.0001 |
| Triglycerides | |  |  |  |  |
|  | Baseline (mg/dL) | 127 (91, 176) | 122 (90, 167) |  |  |
|  | Week 12 (mg/dL) | 126 (90, 179) | 109 (86, 144) | -14 (-18, -9) | <0.0001 |
|  | Absolute change (mg/dL) | 0 (-26, 25) | -9 (-36, 13) | -11 (-15, -7) | <0.0001 |
|  | Percent change | 0 (-19.7, 24.1) | -7.6 (-25.4, 13.5) | -7.7 (-10.5, -4.9) | <0.0001 |
| Apolipoprotein B | |  |  |  |  |
|  | Baseline (mg/dL) | 86 (73, 105) | 85 (73, 102) |  |  |
|  | Week 12 (mg/dL) | 84 (70, 102) | 66 (56, 78) | -18 (-20, -16) | <0.0001 |
|  | Absolute change (mg/dL) | -2 (-12, 8) | -20 (-34, -8) | -18 (-20, -16) | <0.0001 |
|  | Percent change | -1.5 (-13.1, 8.7) | -24.1 (-35.4, -10.5) | -20.7 (-22.4, -19.1) | <0.0001 |
| Non-Lp(a) Apolipoprotein B | |  |  |  |  |
|  | Baseline (nmol/L) | 1471 (1207, 1838) | 1453 (1207, 1789) |  |  |
|  | Week 12 (nmol/L) | 1415 (1161, 1749) | 1091 (926, 1318) | 309 (-275, 343) | <0.0001 |
|  | Absolute change (nmol/L) | -35 (-223, 129) | -344 (-589, -118) | 303 (-333, 273) | <0.0001 |
|  | Percent change | -2.0 (-14.1, 9.7) | -24.3 (-35.9, -9.8) | -20.7 (-22.4, -18.9) | <0.0001 |

Table S5. Lipid and lipoprotein levels at baseline, Week 12, and change in patients treated with placebo or obicetrapib in the BROADWAY study presented as median (interquartile range) for each treatment group and Hodges-Lehmann median of differences confidence interval midpoint (95% CI) for placebo adjusted differences. P-values are from Wilcoxon tests.

**Table S6. Lipids and Lipoproteins in BROOKLYN**

| **Parameter** | | **Placebo**  **(N=115)** | **Obicetrapib**  **(N=200)** | **Placebo Adjusted** | **P Value** |
| --- | --- | --- | --- | --- | --- |
| Low-density lipoprotein cholesterol | |  |  |  |  |
|  | Baseline (mg/dL) | 110 (85, 144) | 112 (89, 144) |  |  |
|  | Week 12 (mg/dL) | 110 (86, 146) | 68 (51, 95) | -42 (-50, -33) | <0.0001 |
|  | Absolute change (mg/dL) | 1 (-10,12) | -43 (-68, -24) | -46 (-52, -39) | <0.0001 |
|  | Percent change | 1.1 (-6.9, 12.8) | -39.6 (-53.6, -24.1) | -40.3 (-45.2, -35.4) | <0.0001 |
| High-density lipoprotein cholesterol | |  |  |  |  |
|  | Baseline (mg/dL) | 50 (38, 60) | 52 (42, 63) |  |  |
|  | Week 12 (mg/dL) | 49 (39, 61) | 119 (105, 135) | 70 (65, 74) | <0.0001 |
|  | Absolute change (mg/dL) | 0 (-4, 5) | 68 (55, 82) | 68 (64, 71) | <0.0001 |
|  | Percent change | 0 (-8.0, 11.1) | 137.4 (98.5, 171.0) | 134.3 (125.2, 143.4) | <0.0001 |
| Triglycerides | |  |  |  |  |
|  | Baseline (mg/dL) | 130 (89, 178) | 119 (77, 161) |  |  |
|  | Week 12 (mg/dL) | 126 (96,172) | 101 (82, 133) | -22 (-33, -10) | 0.0003 |
|  | Absolute change (mg/dL) | 3 (-22, 22) | -5 (-37, 15) | -11 (-20, -2) | 0.02 |
|  | Percent change | 3.4 (-15.9, 21.8) | -5.1 (-26.4, 17.7) | -8.3 (-15.6, -0.9) | 0.03 |
| Apolipoprotein B | |  |  |  |  |
|  | Baseline (mg/dL) | 98 (83, 122) | 100 (84, 124) |  |  |
|  | Week 12 (mg/dL) | 98 (85, 124) | 78 (64, 92) | -23 (-29, -17) | <0.0001 |
|  | Absolute change (mg/dL) | 1 (-7, 13) | -24 (-40, -8) | -27 (-32, -22) | <0.0001 |
|  | Percent change | 1.3 (-7.0, 12.5) | -24.1 (-34.7, -8.6) | -25.5 (-29.3, -21.6) | <0.0001 |
| Non-Lp(a) Apolipoprotein B | |  |  |  |  |
|  | Baseline (nmol/L) | 1654 (1395, 2087) | 1690 (1412, 2165) |  |  |
|  | Week 12 (nmol/L) | 1629 (1450, 2156) | 1311 (1104, 1585) | 380 (-482, 278) | <0.0001 |
|  | Absolute change (nmol/L) | 34 (-127, 229) | -409 (-682, -126) | 464 (-548, 381) | <0.0001 |
|  | Percent change | 1.5 (-8.7, 13.4) | -23.9 (-34.0, -10.5) | -25.4 (-29.6, -21.3) | <0.0001 |

Table S6. Lipid and lipoprotein levels at baseline, Week 12, and change in patients treated with placebo or obicetrapib in the BROOKLYN study presented as median (interquartile range) for each treatment group and Hodges-Lehmann median of differences confidence interval midpoint (95% CI) for placebo adjusted differences. P-values are from Wilcoxon tests.
